# 360. A First-in-Human Study to Assess the Safety, Tolerability, Pharmacokinetics, and Neutralization Profile of Two Long-Acting Anti-SARS-CoV-2 Monoclonal Antibodies

**DOI:** 10.1093/ofid/ofad500.430

**Published:** 2023-11-27

**Authors:** Josephat Asiago, Moetaz Albizem, Holly L Tieu, Kathryn Stecco, Norman Moullan, Craig Fenwick, Kai Lin, Lauren Poffenbarger, Thomas Lengsfeld, David W Liu, Björn M Hock, Rajeev Venkayya, Prakash Bhuyan

**Affiliations:** Aerium Therapeutics Inc , Boston, Massachusetts; Aerium Therapeutics, Boston, Massachusetts; Aerium Therapeutics, Boston, Massachusetts; Aerium Therapeutics, Inc., San Jose, California; Aerium Therapeutics, Boston, Massachusetts; Lausanne University Hospital, Lausanne, Vaud, Switzerland; Aerium Therapeutics, Boston, Massachusetts; Aerium Therapeutics, Boston, Massachusetts; Aerium Therapeutics, Boston, Massachusetts; Aerium Therapeutics, Boston, Massachusetts; Lavaux Biotech Consulting SARL, Yens, Vaud, Switzerland; Aerium Therapeutics Inc., Boston, Massachusetts; Aerium Therapeutics, Boston, Massachusetts

## Abstract

**Background:**

COVID-19 remains a significant risk to the immunodeficient population, which is more vulnerable to severe consequences and less likely to mount a protective immune response following vaccination. Monoclonal antibodies (mAbs) have been effective and safe, but the emergence of omicron and its subvariants has created an unmet need for new broadly protective and long-acting agents.

AER001 and AER002 are human immunoglobulin-G1 under development for pre-exposure prophylaxis and treatment of COVID-19. These mAbs were isolated from persons previously infected and/or vaccinated against SARS-CoV-2. AER001 and AER002 bind to the receptor binding domain of the spike protein on non-overlapping epitopes and compete with angiotensin converting enzyme-2 (ACE-2) to block viral attachment. AER002 showed strong *in vitro* neutralization activity against omicron strains including BA.2.12.1, BA.4 and BA.5.

**Methods:**

The study is a phase I, randomized, double-blind, placebo-controlled, dose escalation study in healthy adults (n=80) with a primary objective to evaluate the safety and tolerability of the combination of AER001 and AER002 or AER002 alone vs placebo, administered intravenous with dose ranging (100 mg to 1200 mg). The secondary objectives are to assess pharmacokinetics (PK)s, anti-drug antibodies (ADA)s, and neutralization activity levels and safety evaluation through 6-months of follow-up.

**Results:**

As of 21 March 2023, enrolment was completed. A total of 154 adverse events (AE)s were reported in 62 subjects. Most frequent AEs were infusion-related site reactions (IRSRs) 20.13% (31/154), headache 16.23% (25/154) and common cold 10.39% (16/154). Most (98.7%) of the reported AEs were grade 1 (mild) severity. There were no serious adverse events (SAE)s or ADAs and 2 non-AE-associated discontinuations. Only 1.3% (2/154) AEs (IRSRs, erythema and tenderness) were rated as possibly related to the investigational products by the principal investigator.

**Conclusion:**

Based upon preliminary data, AER001 and AER002 show adequate safety with no SAEs or ADAs. Complete safety and PK data through 6 months will be presented at the meeting.

**Disclosures:**

**Moetaz Albizem, MD**, Regeneron: Stocks/Bonds **Norman Moullan, PhD**, Aerium Therapeutics: Stocks/Bonds **Kai Lin, PhD**, Aerium Therapeutics: Employee

